# Design of a fixture for precise perpendicular cuts in bone-implant sample preparation

**DOI:** 10.1186/s13104-026-07813-7

**Published:** 2026-04-11

**Authors:** Mihail Genchev, Bianca Riedel, Coralie Nagels, Hagen Schmal, Eva Johanna Kubosch, Maria Carolina Lanzino, Michael Seidenstuecker

**Affiliations:** 1 G.E.R.N. Center of Tissue Replacement, Regeneration & Neogenesis, Department of Orthopedics and Trauma Surgery, Faculty of Medicine, Albert-Ludwigs-University of Freiburg Medical Center, Hugstetter Straße 55, 79106 Freiburg, Germany; 2 Department of Orthopedics and Trauma Surgery, Faculty of Medicine, Medical Center-Albert-Ludwigs-University of Freiburg, Albert-Ludwigs-University of Freiburg, Hugstetter Straße 55, 79106 Freiburg, Germany; 3 Department of Orthopedic Surgery and Traumatology, Odense University Hospital, Odense, 5000 Denmark; 4 Institute for Ceramic Materials and Technologies (IKMT), University of Stuttgart, Stuttgart, Germany

**Keywords:** Perpendicular cutting holder, Push-out test, Bone-implant interface, Specimen preparation, Roundness analysis

## Abstract

Accurate specimen preparation is essential for biomechanical push-out testing of bone–implant interfaces. A common source of error arises when implants such as pins or screws are not cut exactly perpendicular to their axis. This results in oval or irregular cross-sections that compromise the validity of mechanical testing. In this study, we present a newly developed holder designed to ensure perpendicular sectioning of bone-anchored implants. Titanium pins coated with tricalcium phosphate (TCP) and GB14 were implanted in rabbit condyles and prepared for thin-section analysis using the Donath method. The roundness of implant cross-sections was quantitatively assessed with ImageJ (FIJI 1.53t) based on circularity and aspect ratio parameters. Compared with conventional freehand sectioning techniques, which exhibited a roundness of 0.83 ± 0.06 and an aspect ratio of 1.22 ± 0.09, the novel holder achieved markedly improved geometrical accuracy, with a roundness of 0.99 ± 0.002 and an aspect ratio of 1.003 ± 0.002. This difference was statistically significant (*p* < 0.01). These results demonstrate that the device effectively minimizes geometric artifacts during sample preparation, thereby enhancing reproducibility and reliability in subsequent biomechanical testing. Although the validation was limited to rabbit condyles, the holder is a simple and versatile instrument with the potential for application across different implant types and experimental models.

**Supplementary Information:**

The online version contains supplementary material available at 10.1186/s13104-026-07813-7.

## Introduction

In biomechanical push-out tests, which were used to evaluate bone growth around implants such as pins, screws, or coated rods, it is imperative to prepare bone-implant samples with cross sections that are precisely cut at a 90° angle to the implant axis. Problems arise when the implants are not properly aligned parallel to the joint axis during preparation. Misalignment during the cutting process, particularly in cylindrical implants with novel coatings, can result in the implant appearing oval within the bone section, thereby deviating from its cylindrical shape [1]. This misalignment frequently results in the implant tilting during the push-out test, as the implant is no longer aligned in the direction of loading [2]. This resulted in distorted mechanical results and reduced the reliability of the test, a well-documented issue [1, 3, 4]. Bell et al. [5] present a mechanical testing method allowing force alignment exactly perpendicular or parallel to the implant surface, thereby preventing misapplication and improving test reproducibility. Furthermore, Yenaganti et al. [6] and Helgason et al. [7] demonstrated through finite-element analyses that implant placement angle significantly affects bone stress distribution under loading conditions highlighting the critical role of orientation accuracy. Both histomorphometric and biomechanical studies underscore the significance of precise, perpendicular section orientation in ensuring a valid assessment of implant-bone contact and fixation strength. While numerous studies have delineated histological comparisons between micro-CT and two-dimensional histomorphometry for the assessment of osseointegration in meticulous detail (e.g., enhanced precision, but also emphasizing artifact risks) [8], only a limited number explicitly address the mechanical artifacts caused by misalignment during cutting. This gap underscores the necessity for a pragmatic solution: a universal clamping device that guarantees a perpendicular cut irrespective of the implant’s shape or orientation. The implementation of such a device would markedly enhance the reproducibility and precision of biomechanical assessments by ensuring the alignment of the implant in the direction of the applied force, thereby avoiding errors that might arise from tilting.

## Materials and methods

### Implantation of pins

**Fig. 1:**
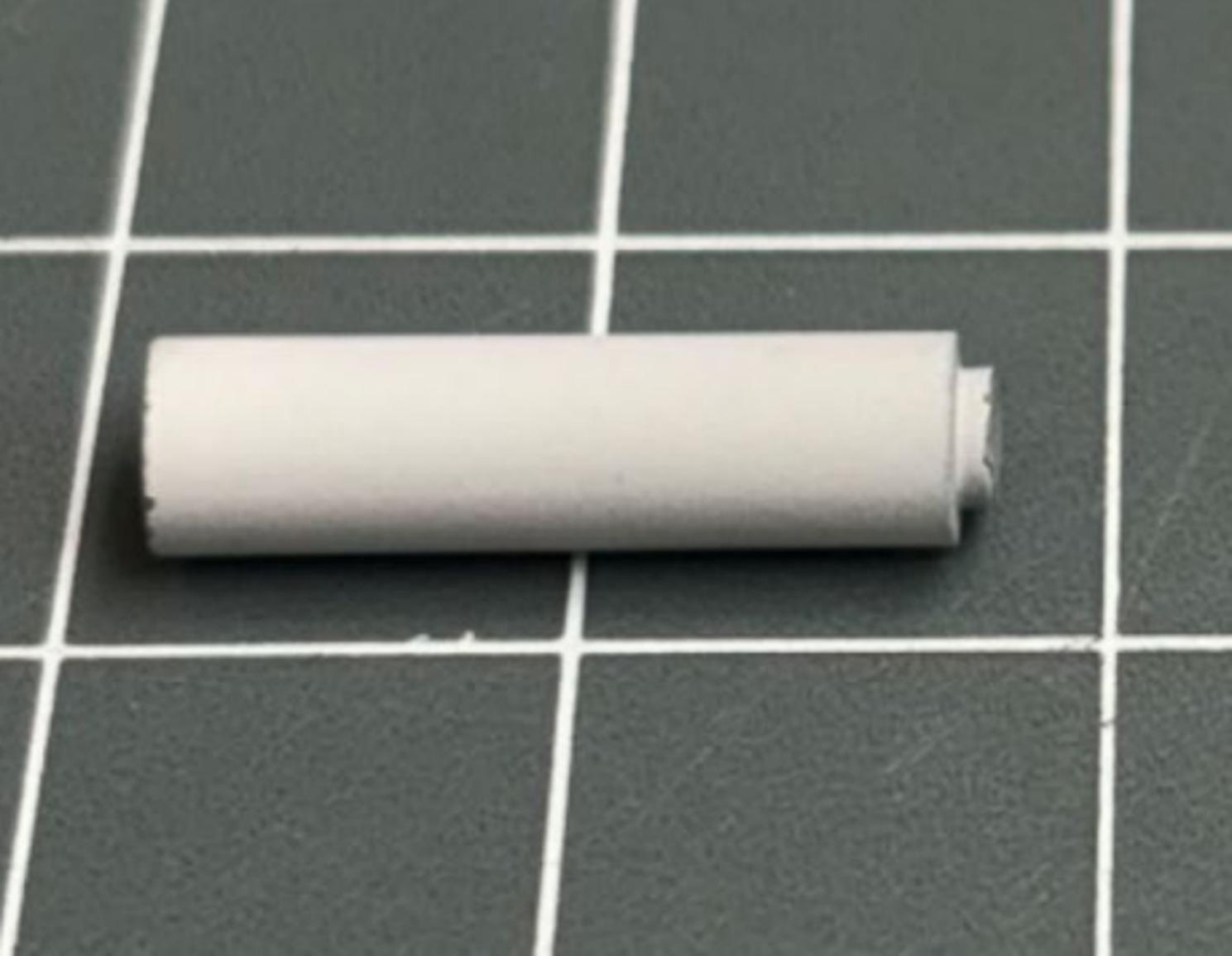
Titanium rod with TCP coating as example; Ø = 4 mm L = 16 mm [14]

The implants used for this work consisted of a titanium pin coated with TCP and GB14 using High Velocity Suspension Flame Spraying [9, 10] (Fig. 1). The coating process and coatings have already been described in detail elsewhere [11, 12], and will not repeated here. After implanting the pins in 21-week-old New Zealand White rabbits (Charles River Laboratories, Écully, France) utilizing a previously described procedure [13, 14], the pins were left in place for 24 weeks to allow for healing. The animals were euthanized after 24 weeks. After anesthesia with 20.0 mg/kg ketamine and 0.3 mg/kg midazolam subcutaneously, euthanasia was induced by the subsequent administration of 5.0–10.0 mg/kg propofol and 2.0 mmol/kg KCl intravenously. The experiment was approved by the Regional Council of Freiburg (Reference number: G-23/079) and carried out in accordance with § 8 TierSchG, Directive 2010/63/EU and ISO EN 10993-2:2023-02 animal welfare requirements [15].

### Sample preparation

**Fig. 2:**
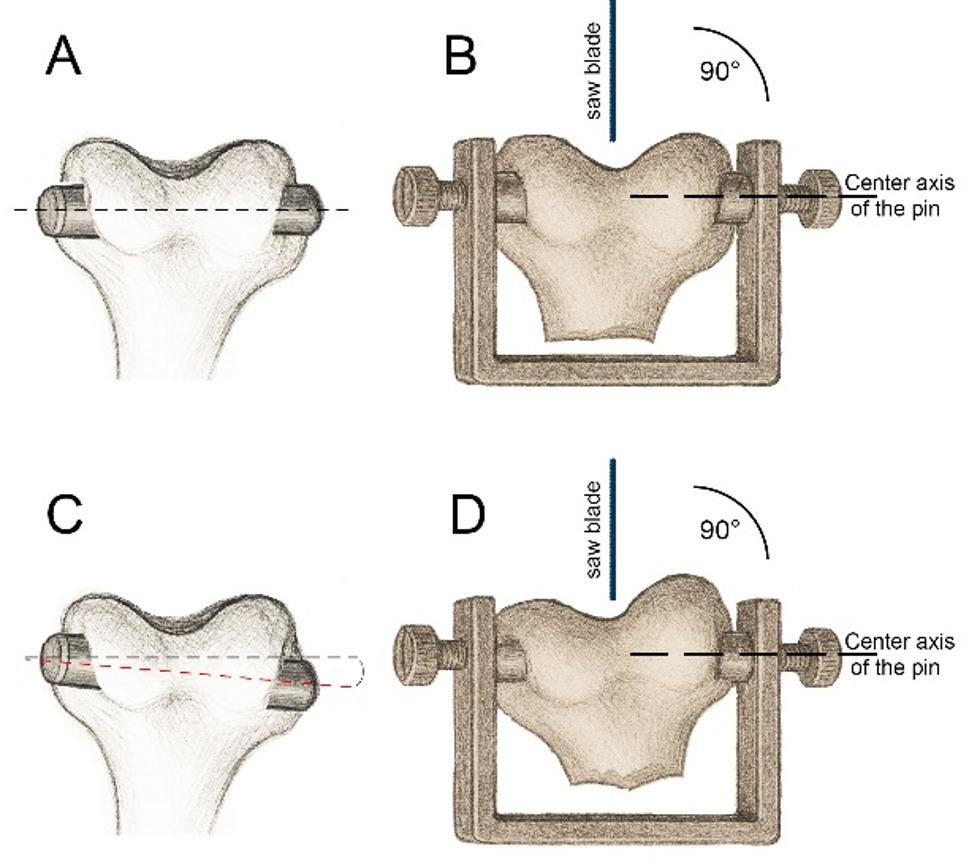
**A**: an optimally implanted pin and **B**: a condyle with an implanted pin clamped in the holder. **C**: a suboptimally implanted pin and **D**: a condyle with an implanted pin clamped in the holder. The saw blade always cuts at a 90° angle to the pin. The pin length has been slightly exaggerated for illustrative purposes

The rabbit condyles were prepared in the same way as Przybilla and Subkov [13, 14] and sectioned through the middle. The only difference was that in the present study the cutting was made with the help of a holding device and not free hand. For the push-out test planned for later, it was essential to saw at a 90° angle to the implant. However, these measurements are not the subject of this paper and have been published elsewhere [14]. The previous freehand procedure consisted of examining the condyles with a C-arm X-ray machine before cutting and marking the position of the pins on the bone with a marker. However, this approach did not result in perfect vertical cuts on the first attempt. Corrective cuts were necessary, which further reduced the already limited sample size (Fig. 2).

**Fig. 3:**
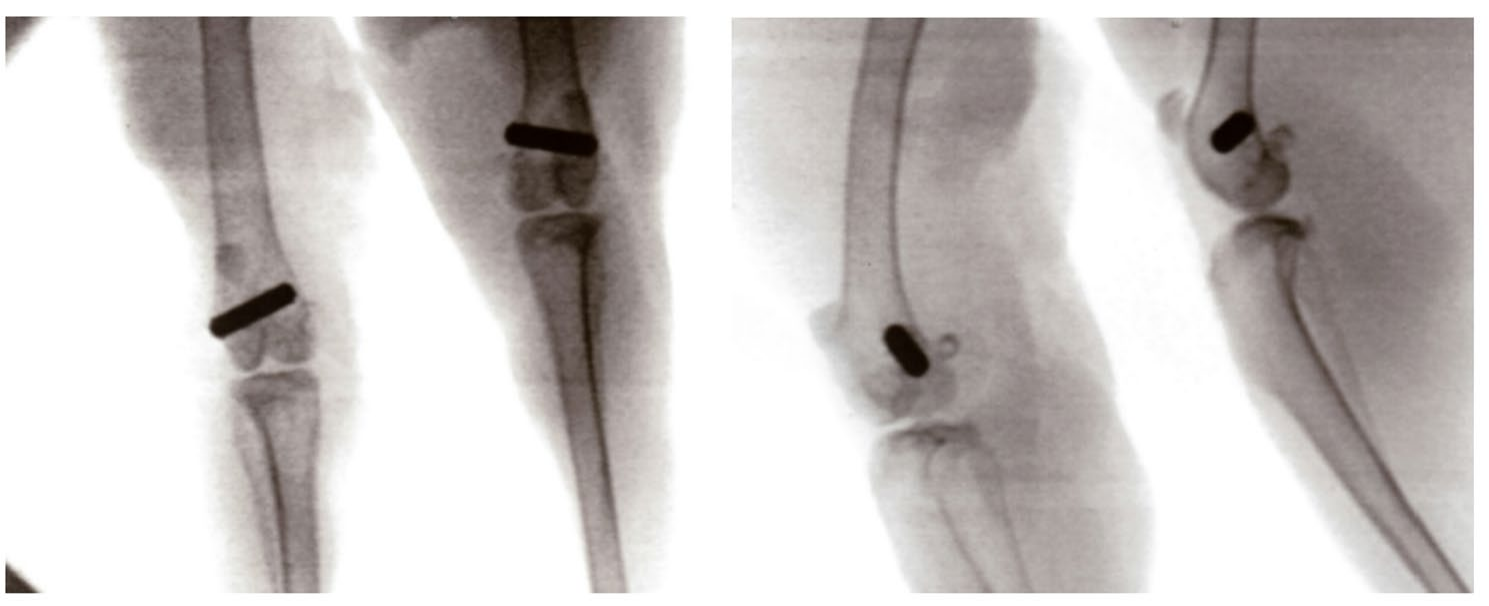
Representative X-ray images illustrating the implant position in two different planes

**Fig. 4:**
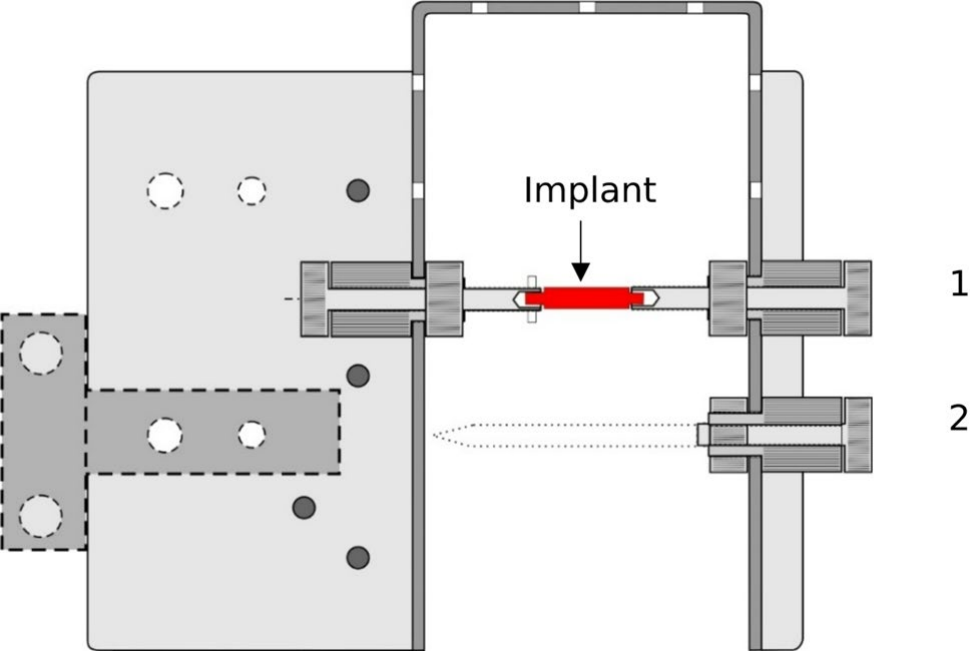
Schematic representation of the holder (pin to be cut in red), illustrating the setup for the first (1) and second (2) steps: (1) Center cut at 90° to the implant; (2) Parallel cuts of the halved samples for defined thicknesses

In the present study, the position of the implant was initially verified using a C-arm X-ray (Fig. 3). Consequently, the implant’s opposing sides were exposed beneath the bone. The bone specimen was subsequently mounted in the designated sample holder. The preparation for the push-out test was executed in two stages. Initially, the condyle was bisected at its midpoint. In the subsequent phase, one half of the specimen was affixed to the opposing wall through the use of a single screw. As illustrated in Fig. 4, the holder is depicted schematically, with the screws designated for the initial and secondary steps clearly visible.

### Technical drawing and schematics

The holder was designed to secure the bone by positioning the implant perpendicular to the saw blade. To achieve this, both ends of the implant, potentially embedded within the bone, had to be exposed (Fig. 4). For improved clarity and reproducibility, schematics of the test setup are provided. In the depicted configuration, the implant had a diameter of 4 mm with two small protrusions of 3.5 mm located on either side. In cases where the implant lacks such protrusions, a temporary auxiliary element may be affixed to the implant and subsequently removed with a scalpel.

### Measurement of roundness

Hard tissue sections containing the implanted pins were prepared according to the Donath method for undecalcified bone [16]. Following dehydration and embedding in light-curing resin, the specimens were cut perpendicular to the implant axis and subsequently ground to thin sections of approximately 250 μm thickness using a precision grinding system. The resulting slides were polished and GIEMSA stained.

Digital micrographs of the implant cross-sections were acquired under transmitted light microscopy (Olympus BX51, magnification 20x). For quantitative assessment of roundness, the images were processed using ImageJ (FIJI modification, Version 1.53t). After calibration to a known scale, the implant cross-section was segmented manually using the thesholding tool to delineate the outer contour. The following geometric parameters were then determined automatically by the software:

- Area (A) of the implant cross-section.
- Perimeter (P) of the contour.
- Circularity (C) = 4π × A / P², with *C = 1* indicating a perfect circle.
- Feret’s diameter (maximal and minimal), representing the longest and shortest distances across the shape.
- Aspect ratio (AR) = Feret_max_ / Feret_min_, describing deviation towards an elliptical geometry.

Circularity values were used as the primary outcome to quantify deviations from ideal roundness. Additional parameters (aspect ratio, Feret diameters) were analyzed to detect systematic elongation or irregular contour formation. All measurements were repeated independently by two investigators, and the mean of both values was used for subsequent analysis. Thin sections from a previous project that addressed a similar question serve as a control [13, 14].

### Statistiscs

All data are reported as mean values accompanied by their respective standard deviations. Prior to statistical testing, the distribution of the data was examined using the Shapiro–Wilk test. For comparisons between groups, a one-way ANOVA with Tukey’s post hoc test was applied, considering differences significant at *p* < 0.05. Statistical processing was carried out with OriginPro 2023 SR1 (OriginLab, Northampton, USA).

## Results

### Development of the mounting bracket

**Fig. 5:**
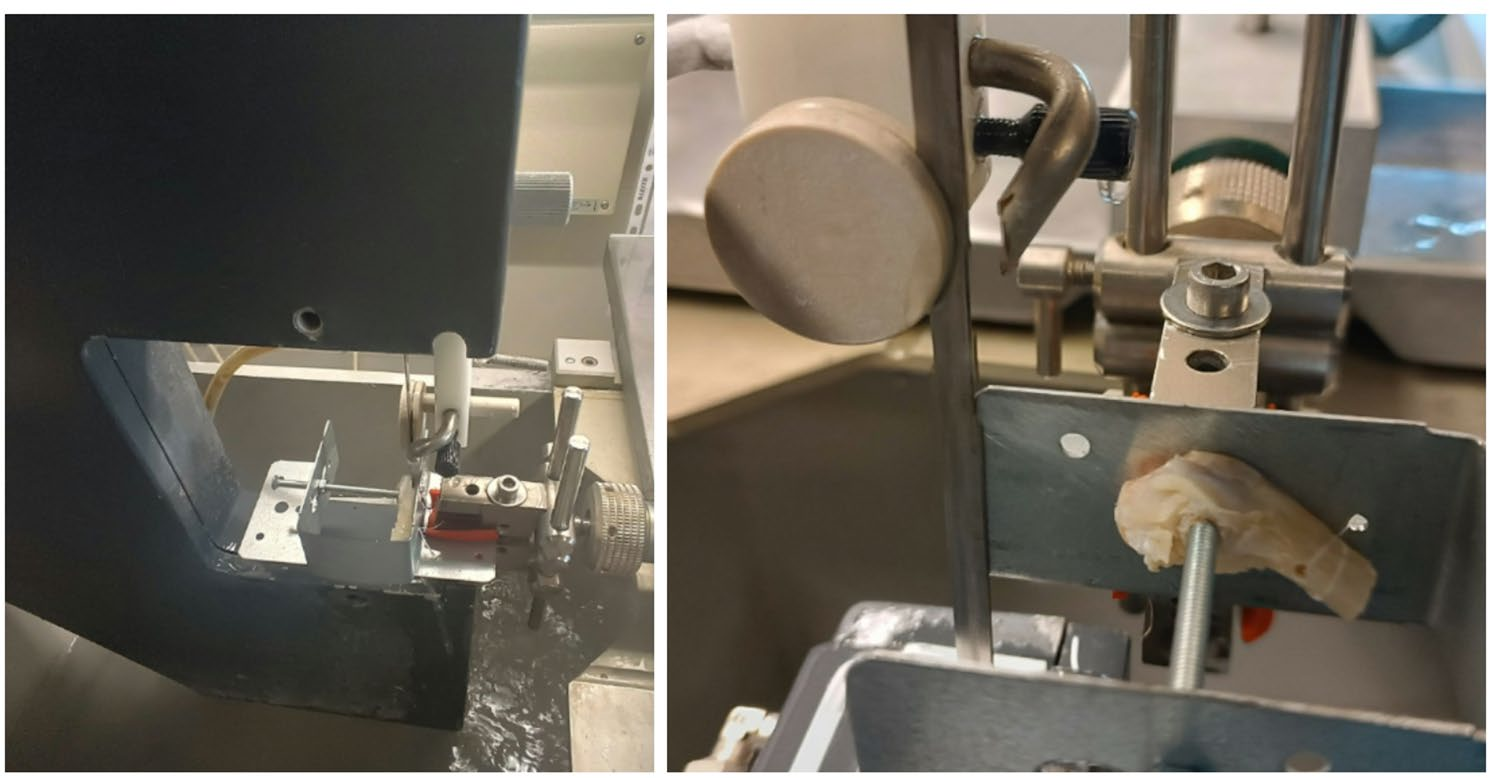
The first design for the holder

The initial design for the holder incorporated a U-shaped frame with two pointed screws on the left and right, which were intended to secure the bones in place. The initial attempt was executed using a U-shaped post bracket designed for square timbers. The bone was clamped into the U-shape, and the bracket was used to clamp it into the band saw (EXAKT Advanced Technologies GmbH, Norderstedt, Germany) (see Fig. 5). However, the sheet metal was found to be of insufficient thickness, resulting in the holder’s tendency to swing, thereby rendering it impossible to make any cuts. With a normal screw nut the screw alignment was insufficient and due to the metal sheet thickness the sheets used to bend out. That led to an unwanted compression of the cutting gap in the process of cutting.

**Fig. 6:**
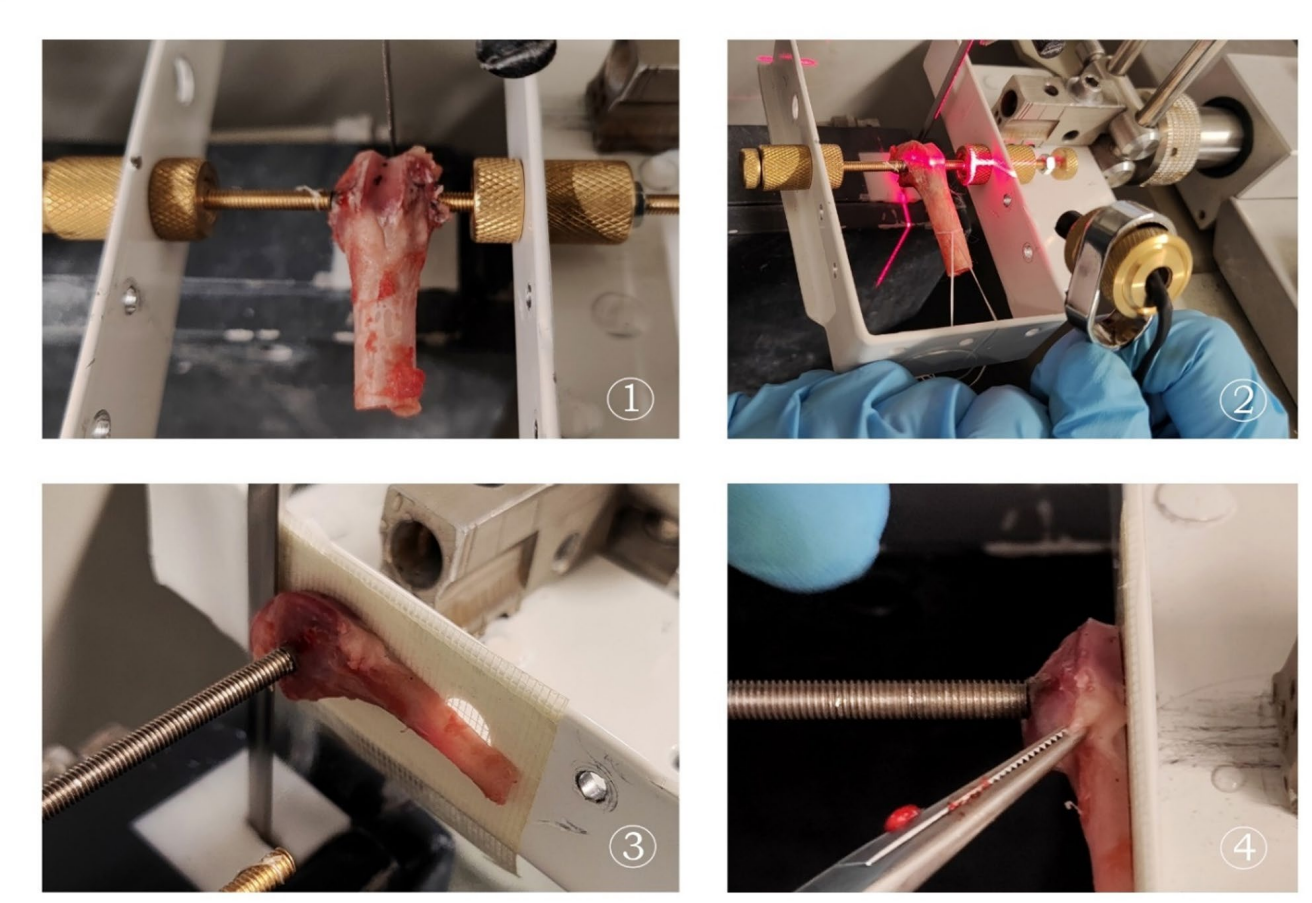
Final design of our holder, which can be used to saw the condyles at a 90° angle to the implant: (1) Position the condyle so that it can be sawed centrally at a 90° angle to the implant; (2) Laser-precise determination of the sawing position; (3, 4) Half condyles fixed at the edge for sawing the 2 mm thick discs, which would be later used for the push out testing

We corrected those two problems with the second version, where we thickened the walls, so they can’t bend and swing from the oscillations of the band saw. We exchanged the screw nut with a longer sleeve to guarantee the screw alignment. An additional laser enabled us to check the desired sawing location in advance and to double check the exact position of the cut (see Fig. 6). To ensure that fixation was achieved solely by the pin and not by interaction with the surrounding bone tissue, screws with a diameter of 4.0 mm were used for the pin with the same diameter. The pin itself had a small protrusion with a diameter of 3.5 mm. The end of screw had an opening with the same diameter, so that they can fit to one another like lock and key. The laser (Fig. 6 − 2) was utilized to preset the planned cut, employing the adjusting screws on the bracket. This procedure can be verified during the sawing process, and adjustments can be made if necessary by altering the setting of the adjusting screws.

**Fig. 7:**
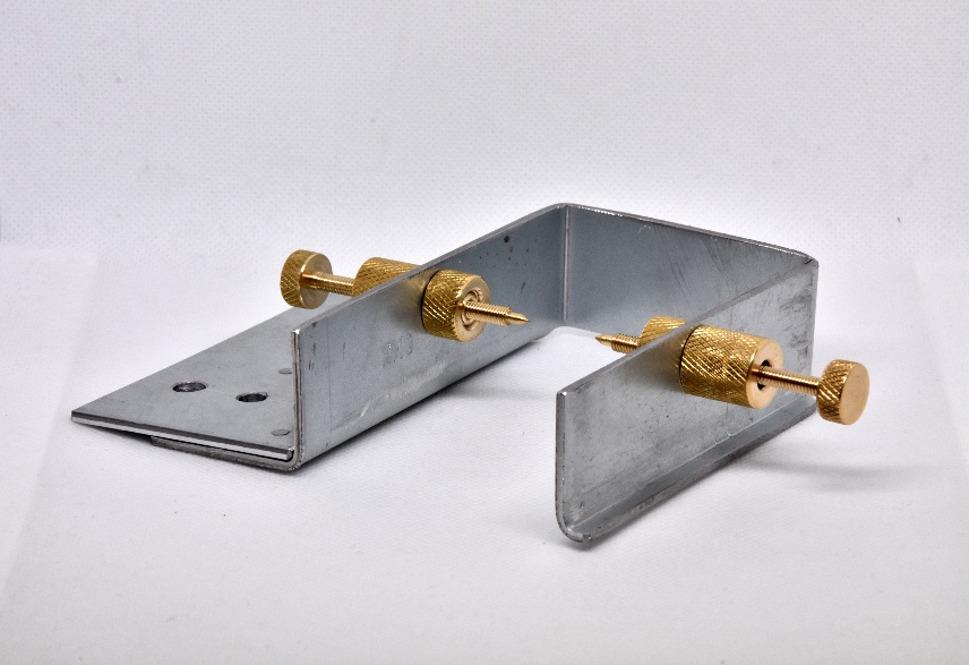
Example of a holding system for fixing the bone itself. The main focus should be on the special screws for a direct screw/bone connection

To check if the clamp can be used to cut a bone with unexposed implant ends, we used another type of screw with a pointy head. It was also used to test the stability of the holding device, so the condyles used in this case had no implant in them (Fig. 7). The connection between the device and the implant depends on the inside part of the screw. An adapter could be used for unconventional implant forms.

### Measurement of roundness

**Fig. 8:**
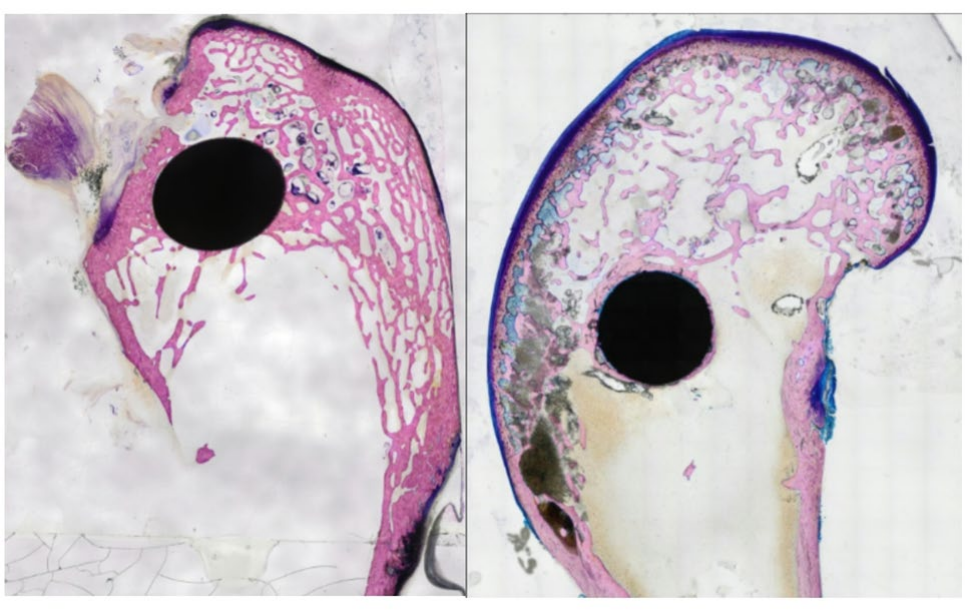
Roundness of the Pin after cutting: left: cut by eye; right: cut with our holder

At first glance, the thin sections stained with GIEMSA clearly show a significant difference between the freehand section cut by eye (Fig. 8, left) and the section cut with our new holder (Fig. 8, right).

**Fig. 9:**
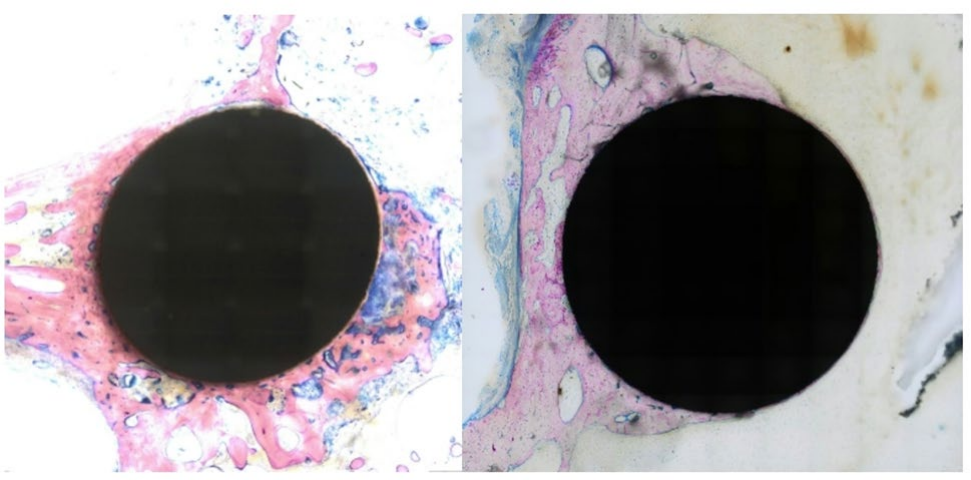
Comparison of the quadratic images used to determine roundness: left previous project; right current project

To facilitate the manipulation of the images, a square image was created with the pin positioned at the center. Subsequently, the samples were examined for roundness employing the ImageJ software. This revealed significant differences in roundness: 0.83 ± 0.06 in the previous project versus 0.99 ± 0.002 in the current project with the new holder. The same holds true for the aspect ratio, which was 1.22 ± 0.09 in the previous project and 1.003 ± 0.002 in the current one. The following Table 1 offers a concise overview of the parameters that were ascertained. Figure 9 provides a visual comparison of the square sections utilized in the GIEMSA stains.

**Table 1:** Comparison of roundness parameters [*n* = 10]

Area [mm²]	Previous project	Current project
	15.145 ± 0.772	12.545 ± 0.177
Circularity	0.826 ± 0.061	0.997 ± 0.002
Feret_max_ [mm]	4.931 ± 0.283	4.048 ± 0.045
Feret_min_ [mm]	4.008 ± 0.086	3.994 ± 0.032
Aspect ratio	1.215 ± 0.086	1.003 ± 0.002

## Discussion

A major methodological challenge in biomechanical push-out testing is the preparation of specimens with accurate geometry. Several studies have highlighted that misalignment between the loading pin and the implant, as well as irregular specimen shapes, can substantially affect the results and increase variability [5, 17]. To address this, various customized fixtures have been described in the literature to ensure coaxial loading [3, 4, 18]. However, no systematic approaches have been reported that specifically guarantee the preparation of cylindrical specimens cut from bone-anchored pins or screws. The fixture developed in this study addresses this limitation by enabling perpendicular cutting of implants within the bone, thereby ensuring high cylindricity of the specimens. This reduces roundness errors, which would otherwise lead to inconsistent load transfer and measurement inaccuracies. Since the pins used did not have a central hole, as was the case with Frosch et al. [1, 3] or Seong et al. [19], for example, to ensure the alignment of the samples via an inserted wire and associated frame, it was necessary to design a new holder. Compared to conventional preparation techniques, the fixture enhances reproducibility and facilitates comparability across different experiments. Thus, this technical innovation represents an important step toward standardization of push-out testing. Our fixture directly addresses these shortcomings by enabling the preparation of standardized cylindrical specimens with precisely perpendicular cut surfaces, thereby reducing errors caused by irregular geometry and misalignment.

It is important to address the following factors for successfully creating a similar holding device. Its structure should be stable and robust so it wouldn’t bend by clamping in the implant or oscillate with the saw itself. The screws should be placed in a longer sleeve so they stay in line. The implant should not be pressed together by the screws. To be able to fulfill those requirements it might be helpful to plan the connection between the implant and the screw in advance. If adjustments are not possible an protruding axillary peace can be glued to the implant to ensure stable connection.

### Limitations

This study has several limitations. First, the newly developed holder was validated exclusively in rabbit condyles. While the concept is theoretically transferable to other small bones, its applicability to larger animal models or human specimens has yet to be evaluated. Second, the size of the U-frame limits the dimensions of the bone samples that can be processed. Consequently, the current setup is limited to smaller bones; larger frame designs are necessary for testing bigger specimens or implants. Third, although the holder was designed to accommodate pins, screws, and wires, systematic validation across different implant types is pending. All experiments were conducted under controlled in vitro conditions. The setup is for now only tested for a bicortical implant. A monocortical one might need adjustments of one of the screws. We acknowledge that this setup might not be best suited for a monocortical implants.

## Conclusion

The newly developed holder precisely prepares bone implant samples by ensuring vertical cuts through pins, screws, or wires implanted in the bone. Conventional cutting methods achieved only 82.6% roundness of the exposed implant cross-sections; however, the use of the holder increased roundness to 99.7%. This high degree of roundness minimizes shear forces during subsequent push-out testing, thereby improving the validity and reproducibility of the results. Despite limitations in current sample size and validation in larger models, the holder is a simple, effective tool for standardizing the preparation of bone for biomechanical and histological testing of bone-implant interfaces.

## Supplementary Information

Below is the link to the electronic supplementary material.

Supplementary Material 1.

## Data Availability

The datasets used and/or analyzed during the current study are available from the corresponding author on reasonable request.
