# Supplementary figures and images for: Design of a fixture for precise perpendicular cuts in bone-implant sample preparation
Source: BMC Res Notes. 2026 Apr 11;19:186. doi: 10.1186/s13104-026-07813-7 (PMC13122900; doi:10.1186/s13104-026-07813-7)

# Positioning sleeve with bolt

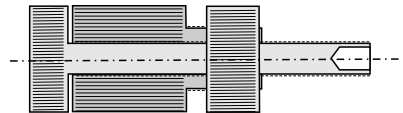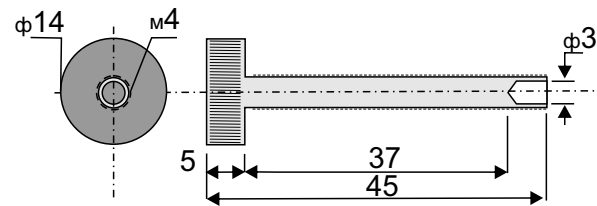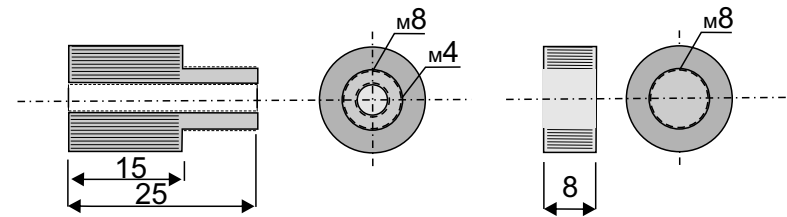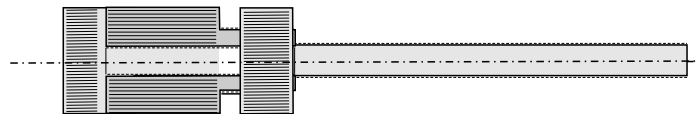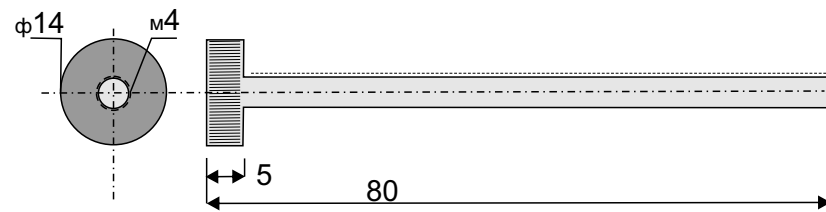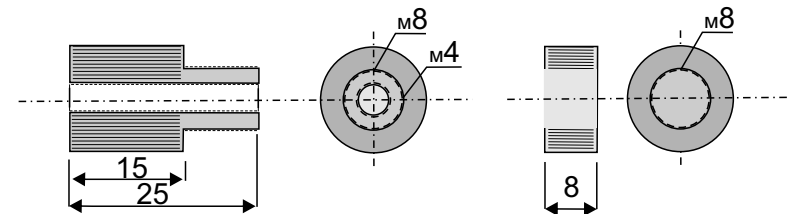

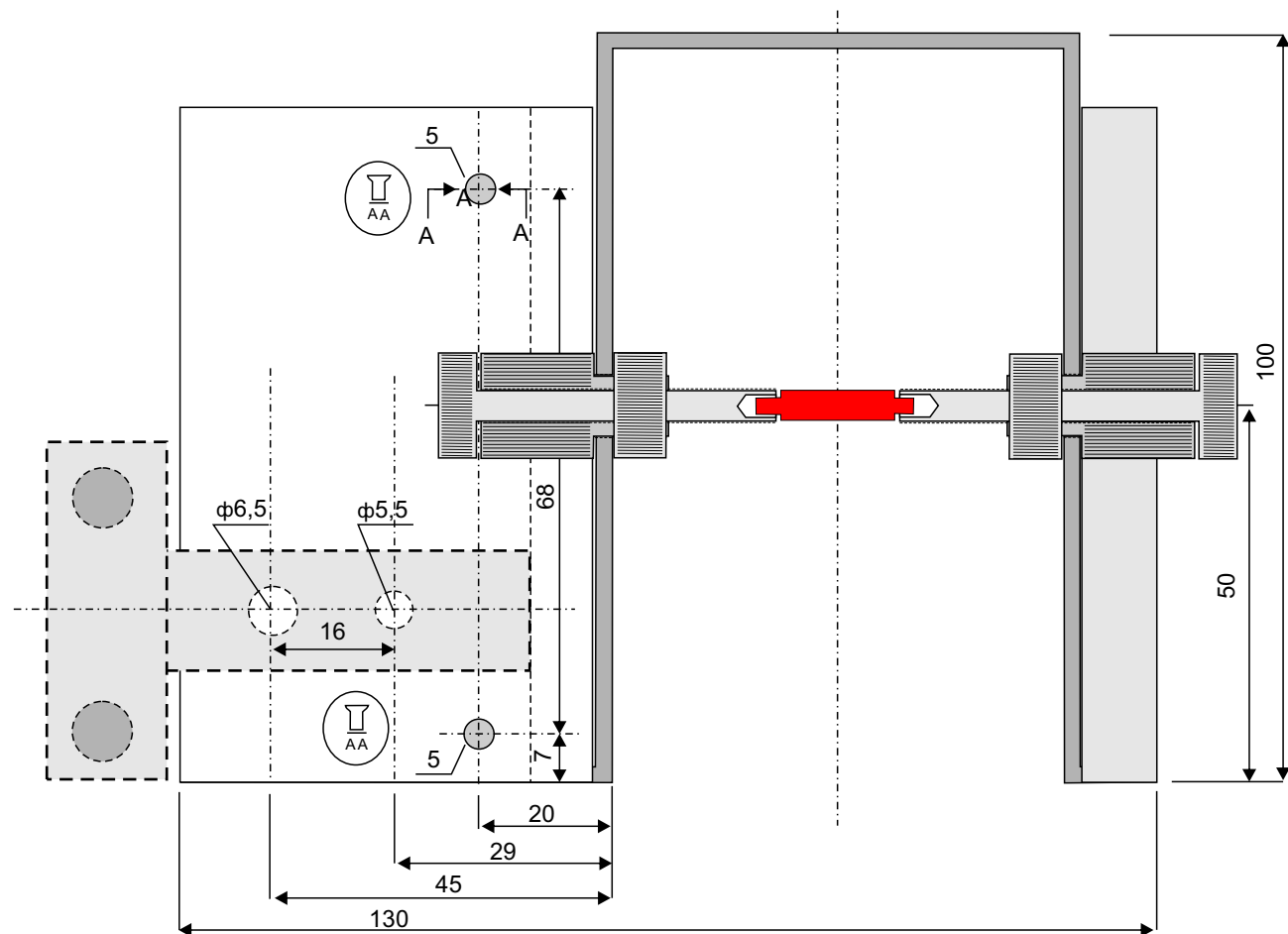

Mihail Genchev  
**Cutting clamp**  
 Construction drawing № 3  
 Freiburg /20.9.2024

Supplement: Supplementary file 1 — Supplementary Material 1. [file 13104_2026_7813_MOESM1_ESM.pdf]
